# The internal representation of fractions: component, holistic or hybrid?

**DOI:** 10.3389/fnins.2026.1705922

**Published:** 2026-03-20

**Authors:** Weimin Lin, Yun Pan, Jun Zhu, Liangzhi Jia, Huanyu Yang, Yajie Bi, Fangwen Yu, Di Zhang

**Affiliations:** 1 School of Psychology, Guizhou Normal University, Guiyang, China; 2 Guizhou Education University, Guiyang, China

**Keywords:** fraction, hybrid representation, shifts of spatial attention, spatial representation, stimulus detection paradigm

## Abstract

This study aimed to differentiate among componential, holistic, and hybrid accounts of fraction representation through three behavioral experiments. Using the stimulus detection paradigm, we systematically tested the competing theoretical hypotheses of spatial attention mapping in fraction processing. Experiments 1 and 2 found that fraction numerical processing could effectively regulate spatial attention under conditions of consistent numerical information. The crucial Experiment 3, which employed a conflict design (where the overall value and component size information were contradictory), did not observe a significant spatial attention effect. Integrating these findings, fraction processing may simultaneously activate both holistic and component information, with its behavioral output being subject to immediate evaluation and regulation; automatic expression is permitted when information is consistent, whereas inhibitory control is triggered in the presence of conflict. The research results reveal the dynamic dual-processing of fractions in the domain of spatial attention. These findings provide key behavioral evidence for understanding the interaction between rapid extraction and controlled processes in mathematical cognition.

## 1 Introduction

Mastering the relevant knowledge of rational numbers is crucial for the development of mathematical abilities (Park and Esposito, 2025; Rojo et al., 2025). The knowledge of fractions constitutes a significant component of rational numbers and holds an important position in mathematics education. Understanding the knowledge of fractions is of significant importance for predicting success in mathematics (Siegler et al., 2012; Schwarzmeier and Obersteiner, 2025). Booth and Newton (2012) suggested that knowledge of fractional concepts may serve as a foundational capability for understanding and mastering advanced mathematical concepts, such as algebra. Fractions, as subsets of real numbers, can be expressed as the quotient of two integers (e.g., 1/5). They are regarded as fundamental components of mathematics education (Ni, 2001). Owing to the close relationship between fractions and integers, individuals often employ integer strategies within a single-unit counting scheme when dealing with fractions. This phenomenon is referred to as “whole number bias” (Ni and Zhou, 2005). The difficulties that children encounter when dealing with and representing fractions can be partly attributed to the interference of whole number information (Braithwaite and Siegler, 2018; Siegler and Lortie-Forgues, 2014). An understanding of the world is fundamentally grounded in numerical concepts; hence, exploring mental representations of numbers is critical (Zeng et al., 2022).

Dehaene et al. (1993) discovered in an experiment on the judgment of digital parity that when participants were faced with smaller digital stimuli, the left hand responded faster; while when dealing with larger numbers, the reaction time (RT) required by the right hand was relatively shorter. This phenomenon is referred to as the spatial-numerical association of response codes (SNARC). Since then, numerous studies across various experimental tasks have identified the SNARC effect, indicating its widespread applicability (Aleotti et al., 2020; Nuerk et al., 2004; Kopiske et al., 2016; Leth-Steensen and Citta, 2016; Fischer and Shaki, 2016). Fischer et al. (2003) conducted an experiment utilizing the Posner's cueing paradigm, which revealed that when the cue numbers were smaller (1 and 2), participants exhibited significantly shorter reaction times for detection targets appearing on the left side. Conversely, when the cue numbers were larger (8 and 9), participants demonstrated notably shorter reaction times for detection targets located on the right side. The present phenomenon expands the scope of the SNARC effect to encompass attentional processes, thus referred to as the attentional SNARC effect. However, Colling et al. (2020) attempted to replicate Fischer et al. (2003) study using a large sample across multiple laboratories; nevertheless, they were unable to observe the attentional SNARC effect. Fischer et al. (2020) indicated that the failure to replicate these findings may be attributed to the neglect of the processing depth associated with digital cues. Recent eye tracking studies have shown that numerical cues can trigger a shift in spatial attention (Myachykov et al., 2016; Salvaggio et al., 2019, 2022). Research on the SNARC effect implies that when individuals engage in the visual processing of numbers, an underlying spatial attention mechanism is activated (Fischer et al., 2003; Hubbard et al., 2005).

The abovementioned studies primarily investigated the relationship between integer processing and spatial representation; however, the effects of fraction processing on the transfer of spatial attention remain ambiguous. There is an ongoing debate concerning the mental representations of fractions. In a fraction comparison task (e.g., 1/3 vs 1/5), Bonato et al. (2007) observed that the distance and SNARC effects were influenced by the fractional components (numerator and denominator) rather than by the real value of the fraction. This suggests that the representation of the fraction is based on its whole number components (numerator and denominator) rather than treating the fraction as a specific quantitative value (holistic representation). Various strategies employed in fraction processing may cause the SNARC effect to disappear or a reverse SNARC effect to emerge (Pan et al., 2023). Notwithstanding, some researchers argue that individuals can directly represent the value of fractions (i.e., a holistic representation). Schneider and Siegler (2010) investigated whether adults use components or holistic representations when comparing fractional values. In the three experiments of the present study, undergraduate participants were assigned the task of comparing fractions with either single or multidigit numerators and denominators, followed by an assessment designed to illustrate the effect of distance. The experimental results show that adults perceive fractions as a whole when comparing their magnitudes. Furthermore, some studies suggest that when comparing fractions, individuals may adopt a hybrid representation strategy (that is, during the processing of fractions, both component processing and holistic processing coexist), and this may depend on task requirements, context, and so on. Meert et al. (2010a) employed a priming paradigm to uncover a potential componential interference effect in children's fraction processing. Specifically, they observed that when children were primed with fraction pairs sharing identical numerators but differing denominators (e.g., 4/7 vs. 4/5), their subsequent responses in natural number comparison tasks (e.g., 7 vs. 5) were slower than in control conditions. This delay suggests interference arising from the automatic activation of magnitude-related representations associated with the denominator during fraction comparison. Such findings imply that a larger denominator may spontaneously evoke a perceptual or conceptual sense of “largeness,” which conflicts with the fraction's overall numerical magnitude—thereby necessitating additional cognitive resources to resolve the conflict and leading to prolonged response times. This finding provides important evidence for understanding how overall and component information compete in fraction processing. In a study examining how adults compare the magnitudes of fractions with no shared components, Meert et al. (2010b) likewise provided evidence consistent with a hybrid representational strategy. Inhibition control is a necessary factor for individual processing of fractions (Rossi et al., 2019). Overall, the representation strategies for fractions remain insufficiently defined. Previous studies suggest that the intrinsic representation of numbers may be influenced by contextual factors (e.g., as task requirements) (Bonato et al., 2007; Mingolo et al., 2021; Prado and Knops, 2024; Tzelgov and Ganor-Stern, 2005; Zaks-Ohayon et al., 2022). Mingolo et al. (2021) found that task requirements may influence mental representation of numbers. Ito and Hatta (2003) noted that individuals tend to develop strategies based on task requirements. To gain a more profound understanding of the fundamental characteristics of mental representations, researchers should employ paradigms that minimize reliance on task demands (Kadosh et al., 2008).

A stimulus detection task, in which irrelevant numerical stimuli can influence the allocation of attention during target detection (Fischer et al., 2003; Dodd et al., 2008), is better suited than a size judgment task to reveal the association between numerical processing and its spatial representation. Moreover, employing a stimulus detection task ensures that fractions are perceived as integrated numerical entities, thereby preventing participants from disregarding any component of the fraction (i.e., numerator or denominator). By adopting this paradigm, researchers can gain deeper insights into the intrinsic representational patterns of numerical magnitude within the domain of attentional spatial processing. In previous studies on fractions, the experimental tasks adopted mostly explicitly required participants to consciously judge the numerical magnitude of fractions (for example, fraction comparison tasks, fraction estimation tasks). Participants' responses may have been influenced by individual strategic processing, which could have confounded the measurement of the intrinsic representation of numbers. Therefore, we argue that employing a stimulus detection task may facilitate a more direct and effective exploration of the mental representations underlying fractional processing. In the domain of numerical cognition, stimulus detection is a well-established and widely used paradigm (Dodd et al., 2008; Fischer et al., 2003; Zeng et al., 2022).

In the present study, we employed a stimulus detection task (Dodd et al., 2008) to investigate the processing of fractions and their association with spatial representations. In each trial, a fraction presented in numerical format (e.g., “1/3”) appeared on the screen. Participants were instructed to ignore the items presented at the fixation point and to respond only when the target stimulus—a black circle—appeared subsequently, by pressing a designated response key. Furthermore, similar to the approach employed by Pellegrino et al. (2021) in their study on the attentional SNARC effect, we extended the prompt duration from 300 ms to 500 ms in the present research's cueing task. We conducted three experiments to investigate whether the processing of fractions can elicit shifts in spatial attention, aiming to explore the representation of fractions.

In previous research (Dehaene et al., 1993), participants performed a parity judgment task on numerical digits and exhibited significantly faster responses with the left hand for small numbers, whereas responses to large numbers were significantly faster with the right hand. Dehaene et al. proposed that during numerical processing and representation, individuals mentally organize numbers along a spatially oriented mental number line (MNL), which is likely oriented from left to right, with smaller numbers represented on the left side and larger numbers on the right. Building upon this body of evidence and theoretical framework, a critical unanswered question remains: although previous research has examined the holistic and componential processing of fractions, the nature of their dynamic interaction at the level of spatial attention remains unclear. This study employed the target detection paradigm as a sensitive behavioral probe, aiming to test the following theoretical hypotheses by systematically manipulating the numerical relations of fractional stimuli: In experiment 1, materials with overall values consistent with component sizes (i.e., larger overall values correspond to larger component values) were used. We predict that a typical attentional SNARC effect will be observed: that is, when the fraction value is smaller, detection of targets on the left side of the screen will be faster; when the value is larger, detection of targets on the right side will be faster. In experiment 2, materials with the same overall value but different component values were used. Since the overall values of the fractions are the same, and this forces (or promotes) the separate processing of their components, we predict that the spatial attentional shift will be driven by the size of the components, thereby generating an attentional SNARC effect based on component size (rather than overall value). Experiments 1 and 2 were conducted under different numerical relationship conditions. The crucial Experiment 3 employed a diagnostic conflict design (e.g., the overall value is large but the component values are small), aiming to directly test the dynamic conflict monitoring hypothesis. The overall and component information are processed in parallel, and their spatial mapping outputs are dynamically regulated by a rapid conflict monitoring system-when the information is consistent, the spatial mapping is expressed; when the information is in conflict, this mapping is actively suppressed. If the spatial effect is eliminated under conflict conditions, it will strongly support a flexible dynamic processing model that includes active inhibition.

## 2 Experiment 1

In Experiment 1, we consistently used the experimental material comprising the overall fractional value and the magnitude of the fractional components (numerator/denominator). We aimed to investigate whether processing fractions induces shifts in spatial attention.

### 2.1 Materials and methods

#### 2.1.1 Participants

The power calculation was conducted using G^*^Power (Faul et al., 2009) to estimate the required sample size for a repeated measures ANOVA under the condition of a medium effect size (effect size = 0.25, α = 0.05, 1 - β = 0.80, correlation among repeated measures = 0.25). The results indicated that a minimum of 15 participants is necessary. Therefore, a total of 27 right-handed undergraduate students (12 men, 15 women; mean age = 20.07 years, SD = 0.62) participated in this study. All participants were right-handed individuals accustomed to reading horizontally from left to right, with normal vision or corrected vision that met normal standards. All participants received appropriate compensation after the experiment was completed.

#### 2.1.2 Materials and equipment

The stimuli in this study were presented on a 15.6-inch Lenovo laptop screen, which had a resolution of 1366 × 768 pixels. We conducted the experiment using E-Prime 2.0 Professional Software. The response was recorded using the spacebar key on a standard keyboard.

University students are likely to possess sufficient experience and capability to represent the magnitude of unit fractions on a number line (Schneider and Siegler, 2010). Therefore, to increase the difficulty and challenge of the experimental materials, we used both single-digit and double-digit fractions. Four fractions (1/2, 1/3, 16/17, 18/19) were presented prior to the target stimuli as cues in Experiment 1. The four numbers comprised two tiers of real values: small (1/2, 1/3) and large (16/17, 18/19). The cue stimulus was presented in black in 48 Times New Roman font.

#### 2.1.3 Design and procedure

**Figure 1:**
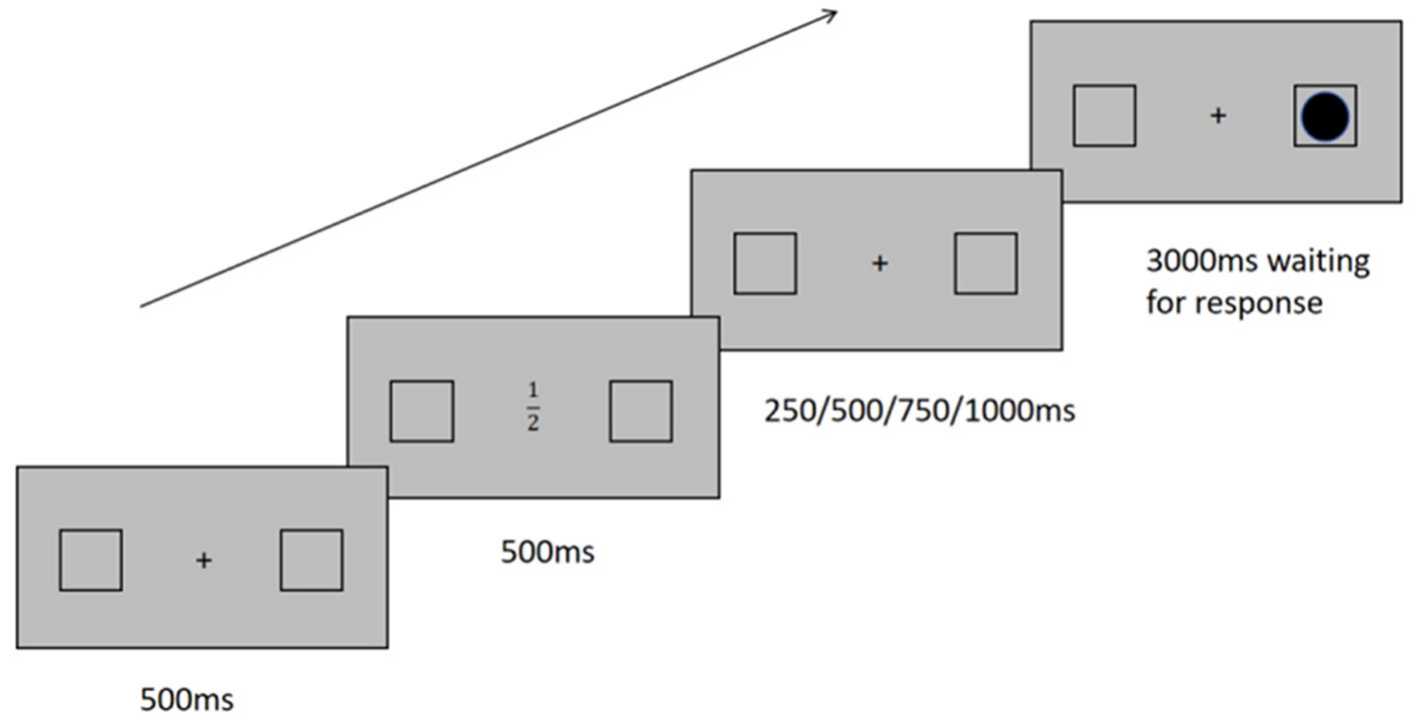
The stimuli detection task. Participants were instructed to promptly press the spacebar upon detecting the target.

In a quiet room, the participants were seated in front of a screen positioned at an approximate distance of 60 cm from their eyes. During the experiment, the participants learned about the response rules of the target-detection task. Subsequently, a central fixation cross (black, with a diameter of 0.5°) and two symmetrically positioned square placeholders (each with a diameter of 2.0° and positioned 4° to the left and right sides of the fixation point) were presented at the center of the screen against a gray background for a duration of 500 ms. Following this, the fixation cross was randomly replaced by one of four pre-selected numbers (i.e., 1/2, 1/3, 16/17, or 18/19). The fixation point was overlaid with one of the four number cues for a duration of 500 ms. Variable cue-target intervals (CTI) of 250, 500, 750, and 1,000 ms were then presented prior to the target stimulus. The target was displayed as a black circle subtended at an angle of 1.8° within one of the two symmetrical placeholder squares. A target presentation, represented by a black circle with an angular size of 1.8°, was displayed within one of the two designated squares for a duration of 3,000 ms or following participants' response. Finally, the trial concluded with a 500 ms blank screen being presented. Participants were instructed to promptly press the spacebar upon detecting the target stimulus. In order to prevent anticipatory responses, the CTI was randomly manipulated across trials (Figure 1).

In the experiment, we employed a within-subjects design with a 2 (target side: left or right) × 2 (number value: small or large) × 4 (CTI: 250, 500, 750, or 1,000 ms). The task involved the identification of target stimuli with utmost precision and speed by pressing the spacebar using the participants' dominant hand. The experiment consisted of 64 practice trials and 480 formal trials. After every 160 trials, the participants were given a brief rest.

#### 2.1.4 Data analysis

Responses with durations less than 100 ms or greater than 1,000 ms were considered as errors and subsequently excluded from further analysis (Dodd et al., 2008). After the data was subjected to logarithmic transformation (i.e., log10(x + 1)) and Winsorization processing, it met the domain standards. We employed a repeated-measures analysis of variance (ANOVA) with the target side (left, right), fractional numbers magnitude (small or large), and CTI (250 ms, 500 ms, 750 ms, or 1000 ms) as within-subjects factors. The dependent variable analyzed in this study was the RT. A significant interaction among target side, number magnitude revealed the presence of an attentional SNARC effect or a reversed effect. Data analysis was conducted using IBM SPSS Statistics version 26, and the calculation of BF_10_ was performed using the JASP statistical package version 0.95.

### 2.2 Results

**Figure 2:**
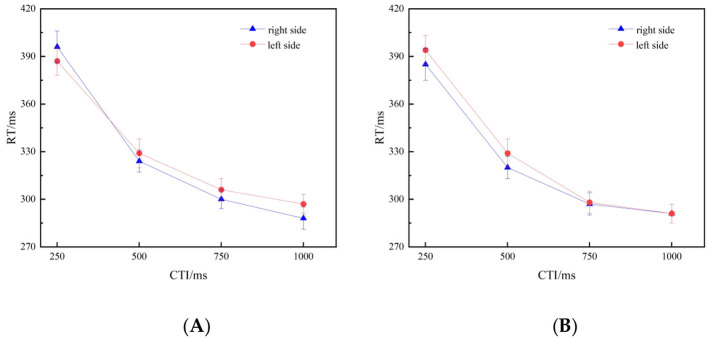
**(A, B)** Illustrate the mean reaction times (RTs) to target stimuli at each CTI. **(A)** Depicts trials preceded by small-magnitude fractions (1/2, 1/3), whereas **(B)** depicts trials preceded by large-magnitude fractions (16/17, 18/19). The corresponding effect sizes (Cohen's d) were as follows: for the small-magnitude fraction condition, d= [−0.44, 0.26, 0.32, 0.35] at 250, 500, 750, and 1,000 ms, respectively; for the large-magnitude fraction condition, d= [0.33, 0.48, 0.04, −0.01]. The error bars represent ±1 standard deviation.

The average error rate was 1.1%; trials with errors were excluded from the analysis. There was a significant main effect of CTI, *F*_(3, 78)_ = 256.158, *p* = 0.000, η^2^*p* = 0.908, indicating that responses were significantly faster at longer CTIs. The other main effects were not statistically significant. Even more importantly, the significant effect was the interaction among the target position, numerical values, and CTI, *F*_(3, 78)_ = 6.221, *p* = 0.001, η^2^*p* = 0.193. Next, we conducted a simple simple effects analysis to identify under which CTI the attentional SNARC effect is present. Specifically, at the 250-ms CTI, the response to the left probe stimulus was faster than that to the right probe stimulus when the small numerical value was presented; in contrast, at the 500-ms CTI, the response to the left probe stimulus was slower than that to the right probe stimulus when the large numerical value was presented. A significant attentional SNARC effect was observed at the 250-ms CTI for small numerical values, *F*_(1, 26)_ = 5.200, *p* = 0.031, η^2^*p* = 0.167, and at the 500-ms CTI for large numerical values, *F*_(1, 26)_ = 6.320, *p* = 0.018, η^2^*p* = 0.196. Further simple effects analysis revealed that when the delay between the numerical cue and the detection stimulus was 250 ms, participants presented with small-magnitude fractions (1/2, 1/3) exhibited significantly faster responses to stimuli on the left side (387 ms) than to those on the right side (396 ms). When the CTI was 500 ms, participants presented with large-magnitude fractions (16/17, 18/19) showed significantly slower responses to stimuli on the left side (329 ms) compared to those on the right side (320 ms). We observed no significant interaction effects for the other delay times (Figure 2). Furthermore, we observed that there was no significant gender difference in the intensity of attentional SNARC effect, *t* (25) = 0.499, *p* = 0.622, Cohen's *d* = 0.193.

To further dissect this interaction and quantify the differences under each specific condition, we conducted paired-samples *t*-tests on the reaction times to the left and right targets at four CTI levels, respectively, under the small-fraction and large-fraction conditions. At a 250 ms CTI in the small-fraction condition, the reaction time to the left target was significantly faster than that to the right target, *t* (26) = −2.28, *p* = 0.031, Cohen's d = −0.44. At a 500 ms CTI in the large-fraction condition, the reaction time to the left target was significantly slower than that to the right target, *t* (26) = 2.51, *p* = 0.018, Cohen's d = 0.48. This result is consistent with the previously observed simple effect pattern. In the other six comparisons, the differences in reaction times between the left and right targets were not significant. Detailed statistical results are as follows: Small-magnitude fractions: 500 ms, *t* (26) = 1.33, *p* = 0.194, Cohen's d = 0.26; 750 ms, *t* (26) = 1.64, *p* = 0.113, Cohen's d = 0.32; 1,000 ms, *t* (26) = 1.81, *p* = 0.081, Cohen's d = 0.35. Large-magnitude fractions: 250 ms, *t* (26) = 1.74, *p* = 0.094, Cohen's d = 0.33; 750 ms, *t* (26) = 0.22, *p* = 0.827, Cohen's d = 0.04; 1,000 ms, *t* (26) = −0.05, *p* = 0.961, Cohen's d = −0.01.

### 2.3 Discussion

The results of Experiment 1 indicate that there is an association between numerical processing and spatial representation, and the processing of numerical magnitude can induce a transfer of spatial attention. This finding indicates that there was an mental number line representing the number value of the fraction in the participants' mental representations. At 250 ms, when the fraction was small, and at 500 ms, when the fraction was large, the reaction time difference to the probe stimulus on the left and right sides was significant, which is consistent with previous findings (Dodd et al., 2008; Galfano et al., 2006; Zeng et al., 2022).

In our experiment, we observed that the small fraction effect reversed after 500 ms and the large fraction effect disappeared after 750 ms, presenting such a temporal dynamic pattern. Therefore, we argue that the early-stage attentional SNARC effect of fractions indicates that their numerical values can be rapidly extracted and mapped onto spatial attention. The emergence of the fraction reversal effect in the mid-transition stage may reflect the involvement of task-induced cognitive control strategies. To optimize overall performance, the system may actively implement an inhibition or attentional shift mechanism, adjusting attentional resources from the direction indicated by the cue to the opposite direction, thereby leading to a reversal in behavioral patterns. In the later stage, as the interval between the cue and the target further increases, any spatial attention advantage triggered by the cue gradually diminishes. Therefore, the spatial attention mapping induced by fractions is not fixed but may be a dynamic process.

Based on the above conclusions, it cannot be inferred that fraction processing induces a spatial attentional shift in accordance with the numerical magnitude of the fractions. This is because previous studies (Meert et al., 2009, 2010a) have shown that the processing of fractions may also activate representations of their component parts (numerator/denominator). In the experimental materials used in Experiment 1, the overall magnitude of each fraction was congruent with that of its corresponding components (numerator and denominator). Therefore, it remains indeterminate whether the shift in spatial attention was driven by the overall magnitude of the fraction itself or by the concurrent activation of its constituent components. As such, the next aspect we wish to explore is whether the components of the fractions, namely the numerator and denominator, are activated during fraction processing.

## 3 Experiment 2

In Experiment 2, we utilized fractions with equivalent overall values but differing component magnitudes as experimental materials (e.g., 1/2, 8/16). Our purpose was to further validate whether the magnitudes of a fraction's components (i.e., the numerator and denominator) were activated during processing and representation of the fractions.

### 3.1 Materials and methods

#### 3.1.1 Participants

The sample size was estimated using G^*^Power (Faul et al., 2009), following the same methodology as in Experiment 1. A different set of 28 right-handed undergraduate students (12 men, 16 women; mean age = 20.11 years, SD = 0.83) voluntarily participated. All participants were right-handed individuals accustomed to reading horizontally from left to right, with normal vision or corrected vision that met normal standards. All participants received appropriate compensation after the experiment was completed.

#### 3.1.2 Materials and equipment

The experimental setup utilized in Experiment 2 was identical to that employed in Experiment 1.

The target stimuli in Experiment 2 were preceded by four fractions (1/2, 1/3, 8/16, and 6/18) serving as cues. The four numbers contained two levels of fractional components: small (1/2, 1/3) and large (8/16, 6/18).

#### 3.1.3 Design and procedure

The procedure and design were replicated from Experiment 1, with the exception that a distinct set of numbers (i.e., 1/2, 1/3, 8/16, and 6/18) replaced the cue stimulus. The study employed a within-subjects design following a 2 (target side: left or right) × 2 (number of components: small or large) × 4 (CTI: 250, 500, 750, or 1,000 ms) factorial structure.

### 3.2 Results

The error rate across all trials was < 2%; we excluded erroneous trials from subsequent analyses. The repeated measures ANOVA revealed a significant main effect for the CTI, *F*_(3, 81)_ = 232.470, *p* = 0.000, η^2^*p* = 0.896. However, no statistically significant main effects were observed for other variables.

**Figure 3:**
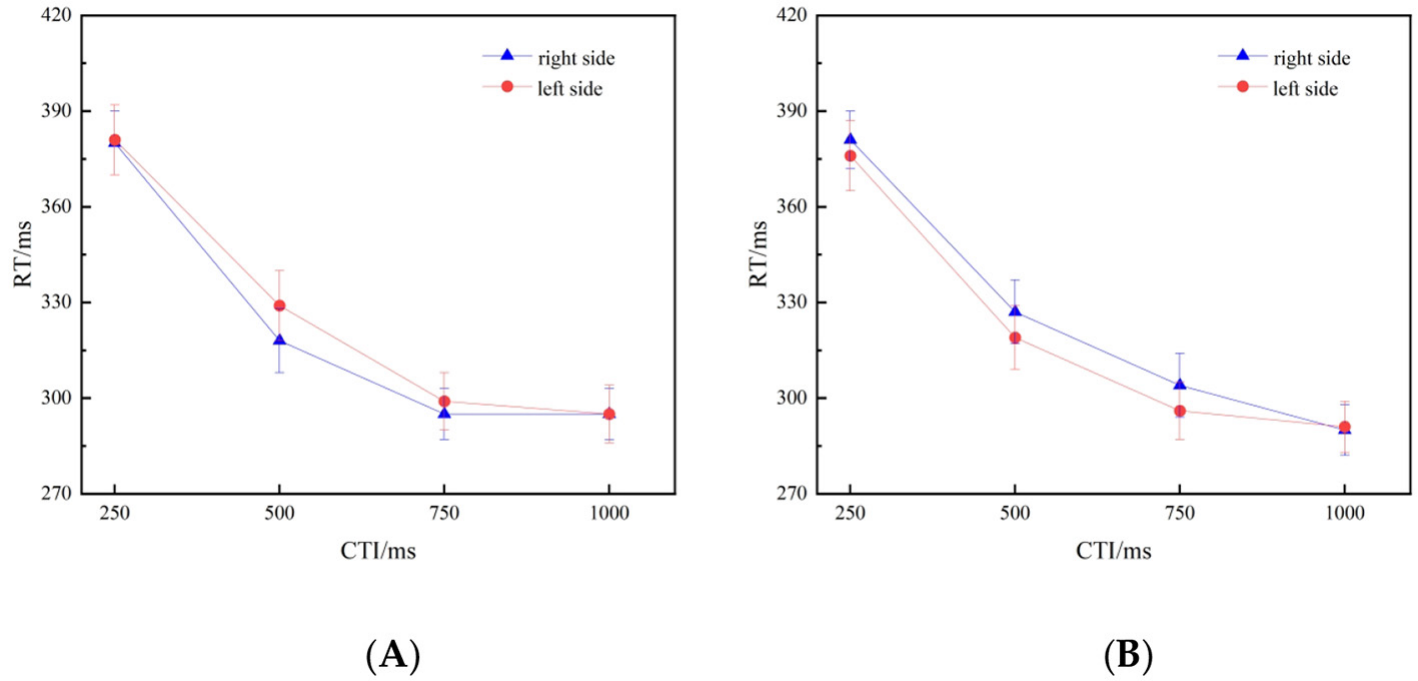
**(A, B)** Illustrate the mean reaction times (RTs) to target stimuli at each CTI. **(A)** Depicts trials preceded by large fractional component (8/16, 6/18), whereas **(B)** depicts trials preceded by small fractional component (1/2, 1/3). The corresponding effect sizes (Cohen's d) were as follows: for the large fractional component condition, d= [0.05, 0.49, 0.15, −0.01] at 250, 500, 750, and 1,000 ms, respectively; for the small fractional component condition, d= [-0.15,−0.33,−0.39, 0.06]. The error bars represent ±1 standard deviation.

We observed a significant interaction between the target side and numerical values, *F*_(1, 27)_ = 9.079, *p* = 0.006, η^2^*p* = 0.252. In a more critical aspect, the significant three-way interaction involved the target position, numerical values, and CTI, *F*_(3, 81)_ = 3.058, *p* = 0.033, η^2^*p* = 0.102. We conducted a simple simple effects analysis to determine the critical time intervals (CTIs) at which the effect was observed. Specifically, we observed attentional SNARC effect at the 500ms and 750 ms CTI. That is, at a 500-ms CTI, when the larger fractional component is presented, the response to the left probe stimulus is slower than that to the right probe stimulus. At a 750-ms CTI, when the smaller fractional component is presented, the response to the left probe stimulus is faster than that to the right probe stimulus. Further analysis of simple effects revealed that when the CTI was set to 500 ms and the cue number represented a larger fractional component (i.e., 8/16, 6/18), participants demonstrated a significantly slower response to stimuli presented on the left side (329ms) compared to their reaction time for stimuli on the right side (318ms), *F*_(1, 27)_ = 6.813, *p* = 0.015, η^2^*p* = 0.202. Additionally, When the CTI was set to 750 ms and the cue number was a small fractional component (i.e., 1/2, 1/3), participants exhibited a significantly faster response to stimuli presented on the left side (296 ms) than to their RT for stimuli on the right side (304 ms), *F*_(1, 27)_ = 4.312, *p* = 0.047, η^2^*p* = 0.138. We observed no significant differences for any of the other CTIs (Figure 3). In addition, similar to the results of Experiment 1, we also observed no significant gender differences in the attentional SNARC effect intensity, *t* (26) = 0.514, *p* = 0.611, Cohen's d = 0.196.

To further dissect this interaction and quantify the differences under each specific condition, we conducted paired-samples *t*-tests on the reaction times to the left and right targets at four CTI levels, respectively, under the small-fraction component and large-fraction component conditions. At a 500 ms CTI in the large-fraction component condition, the reaction time to the left target was significantly slower than that to the right target, *t* (27) = 2.61, *p* = 0.015, Cohen's d = 0.49. At a 750 ms CTI in the small-fraction component condition, the reaction time to the left target was significantly faster than that to the right target, *t* (27) = −2.08, *p* = 0.047, Cohen's d = −0.39. This result is consistent with the previously observed simple effect pattern. In the other six comparisons, the differences in reaction times between the left and right targets were not significant. Detailed statistical results are as follows: Small-fraction component: 250 ms, *t* (27) = −0.78, *p* = 0.441, Cohen's d = −0.15; 500 ms, *t* (26) = −1.73, *p* = 0.095, Cohen's *d* = −0.33; 1,000 ms, *t* (27) = 0.30, *p* = 0.769, Cohen's d =0.06. Large-fraction component: 250 ms, *t* (27) = 0.26, *p* = 0.799, Cohen's d = 0.05; 750 ms, *t* (27) = 0.82, *p* = 0.419, Cohen's d = 0.15; 1,000 ms, *t* (27) = −0.05, *p* =0.964, Cohen's d = −0.01.

### 3.3 Discussion

The findings from Experiment 2 demonstrate that a significant attentional SNARC effect was observed for the magnitude representation of fractions at CTIs of 500 ms and 750 ms. These results are consistent with those of Experiment 1, indicating that the components of the fractions (i.e., the numerator and denominator) can similarly elicit an attentional SNARC effect. However, in contrast to Experiment 1, the attentional SNARC effect was observed in Experiment 2 when the CTI was at 500 ms and 750 ms, implying that the processing of numerical materials in Experiment 2 required a longer duration for spatial attention shifts. This also denotes that more complex numerical judgment processing may require additional attentional resources. We believe that the spatial coding of component size does not emerge in the early stage but only becomes apparent in the middle stage of processing and eventually disappears in the later stage. When the overall value of rapidly presented fractions is evaluated as “no informative difference,” within the limited time window available, the cognitive system may unconsciously establish a temporary connection: associating “processing large number components” with right-sided attention and “processing small number components” with left-sided attention. The disappearance of this effect in the later stage reveals the dynamic nature and resource dependence of this strategy. Once the maintenance time of attention is too long or the task requirements change, this temporary connection will quickly dissolve. This also indicates that the spatial representation of fractions may be a multi-stage dynamic pattern.

At this juncture, it can be deduced that the activation of the components of a fraction (i.e., numerator and denominator) occurs when representing its magnitude. However, as we used two sets of equally numbered but differentially expressed fractional cue lines, we were unable to ascertain whether the overall fractional values were also activated under these experimental conditions. As such, we explored whether both a fraction's overall value and its constituent parts (i.e., the numerator and denominator) were activated simultaneously when representing the magnitude of the fractions.

## 4 Experiment 3

In Experiment 3, the materials presented a conflict between the overall value and the magnitude of their components, such as 2/3 and 8/16. We aimed to investigate whether both the overall value and magnitude of the components were simultaneously activated when processing fractions.

### 4.1 Materials and methods

#### 4.1.1 Participants

The sample size was estimated using G^*^Power (Faul et al., 2009), following the same methodology as in Experiment 1, 2. A separate set of 29 undergraduate students (13 men, 16 women; mean age = 20.17 years, SD = 0.85) voluntarily took part. All participants were right-handed individuals with a habitual left-to-right horizontal reading pattern and normal or corrected-to-normal visual acuity. All participants received appropriate compensation after the experiment was completed.

#### 4.1.2 Materials and equipment

The experimental setup employed in Experiment 3 was identical to that utilized in Experiment 1, 2.

The four fractions (2/3, 3/4, 8/16, 6/18) were presented as preceding target stimuli in Experiment 3. The four numbers contained two levels of the fraction's overall value: small (2/3, 3/4) and large (8/16, 6/18).

The stimulus selection in the three experiments of this study was theory-driven. The specific fractions chosen (such as 8/16 in Experiment 3 of this study) were intended to maximize the separation and manipulation of the core theoretical variables of our concern (such as the conflict between the overall value and the component size). This focused strategy was adopted to efficiently test the existence of the core mechanism at the initial stage of the study.

#### 4.1.3 Design and procedure

The procedure and design were identical to those employed in Experiment 1, with the exception that the cue stimulus was substituted with a distinct set of numerical values (2/3, 3/4, 8/16, and 6/18). The study employed a within-subjects design with a 2 (target side: left, right) × 2 (number value: small or large) × 4 (CTI: 250, 500, 750, or 1,000 ms) factorial structure.

Experiment 3 aimed to create a diagnostic situation to examine how the attentional SNARC effect changes when the overall value of a fraction and the size information of its components may conflict in spatial direction. The selected fractions (such as 2/3 and 8/16) have the feature that their overall values and the absolute values of their numerators/denominators convey opposite spatial cues. This design enables us to test the different predictions of the pure spatial mapping model (overall or component) and the dynamic control model.

### 4.2 Results

The error rate across all trials was < 3%; we excluded trials in which errors occurred from the analysis. The repeated measures ANOVA revealed a significant main effect for the CTI, *F*_(3, 84)_ = 214.618, *p* =0.000, η^2^*p* = 0.885. However, no statistically significant main effects were observed for other variables.

The three-way interaction involving the target position, numerical values, and CTI did not show any significant effects, *F*_(3, 84)_ = 0.577, *p* = 0.632, η^2^*p* = 0.020. To further quantify the strength of evidence supporting the null hypothesis (i.e., no interaction), we calculated the Bayes factor (BF10). The result was BF10 = 0.117. This indicates that the shift in spatial attention was not influenced by a cue number. We observed no significant impacts of other main effects or interactions. Furthermore, consistent with the findings of Experiments 1 and 2, our results also revealed no statistically significant differences in the intensity of the attentional SNARC effect between genders, *t* (27) = 0.599, *p* = 0.554, Cohen's d = 0.228.

**Figure 4:**
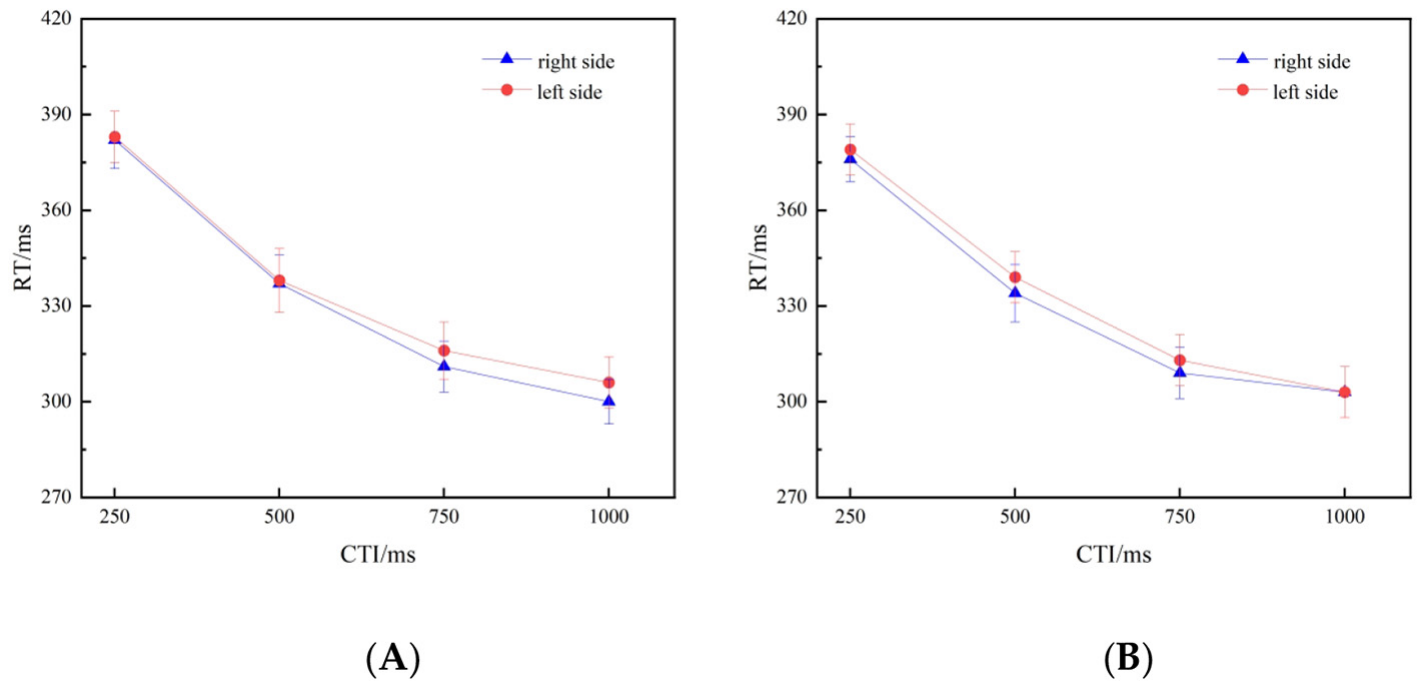
**(A, B)** Illustrate the mean reaction times (RTs) to target stimuli at each CTI. **(A)** depicts trials preceded by large-magnitude fractions (2/3, 3/4), whereas **(B)** depicts trials preceded by small-magnitude fractions (8/16, 6/18). The corresponding effect sizes (Cohen's d) were as follows: for the large-magnitude fraction condition, d= [0.04, 0.03, 0.21, 0.21] at 250, 500, 750, and 1,000 ms, respectively; for the small-magnitude fraction condition, d= [0.14, 0.29, 0.25, −0.02]. The error bars represent ±1 standard deviation.

This experiment employed a conflict design to investigate the modulation pattern of spatial attention when the overall value of a fraction is in opposition to the size information of its components (e.g., a large overall value but small component values, or a small overall value but large component values). Under this conflict condition, the reaction time curves of the left and right targets at each CTI level showed a closely parallel pattern, without demonstrating a systematic separation based on numerical magnitude (Figure 4). The critical three-way interaction was not significant (*p* = 0.632), and the Bayes factor (BF10 = 0.117) provided substantial evidence for the absence of an interaction. This result indicates that when there is a conflict between the overall representation of a fraction and the local component information, the spatial attentional orienting effect automatically triggered by numerical values is eliminated.

### 4.3 Discussion

The findings from Experiment 3 demonstrate that, contrary to the results obtained in Experiments 1 and 2, the processing of fraction magnitude did not elicit a spatial attention shift. Specifically, the conflict arising from the discrepancy between a fraction's overall value and its constituent magnitudes results in the inability to elicit the attentional SNARC effect during processing and representation. In the context of Experiment 3, if the participants focused solely on the magnitude of the fractions, we anticipated that the results would exhibit the classic attentional SNARC effect. This implies that we would obtain an MNL analogous to that formed in Experiment 1; this MNL would be arranged according to the actual numerical values of the fractions. Conversely, if one focuses solely on the components of the fractions (i.e., the numerator and denominator), we expect a reverse attentional SNARC effect.

The results of Experiment 3, which seemed negative at first glance, actually provided crucial insights into the flexibility of fraction representation. Under the condition where the overall value conflicted with the component size information, the predicted automatic spatial attention effect based on any single component did not emerge. Instead, the reaction time curves were completely parallel. This “complete zero effect” pattern, combined with strong Bayesian evidence, ruled out simple explanations such as “automatic activation failure” or “insufficient statistical power.” We believe that the most reasonable explanation is that the conflict triggered active cognitive control. When the cognitive system processes both the overall value of the fraction and the size of the components simultaneously and detects possible contradictory signals they may convey in spatial mapping (such as “the whole is large” suggesting the right side, while “the component is small” suggesting the left side), an immediate conflict monitoring-inhibition mechanism is activated. The function of this mechanism is not to prevent the processing of conflict information, but to prevent these conflicting and task-irrelevant information from further automatically transforming into spatial attention bias, thereby avoiding interference with the current core task (target detection). The “disappearance” of the behavioral effect we observed is precisely the manifestation of this advanced inhibitory control taking effect. Therefore, Experiment 3, together with Experiments 1 and 2, jointly depict a complete model of fraction space processing: In a situation where information is consistent and there is no conflict, the rapid extraction of numerical processing dominates the early spatial attention orientation; while when internal representations conflict, a dynamic, top-down control process quickly intervenes to inhibit the automatic mapping, ensuring the adaptability and efficiency of cognitive operations. This strongly proves that the spatial encoding of fractions is far from a rigid stimulus-response association, but rather a context-regulated dynamic system that includes both rapid extraction and active control processes.

## 5 General discussion

We employed different numerical sets as cue stimuli using a stimulus detection paradigm (Dodd et al., 2008). We aimed to investigate whether fraction processing is associated with spatial representation and whether it can elicit a shift in spatial attention. The results of Experiment 1 and Experiment 2 indicate that the processing and representation of fractions can trigger a shift in spatial attention; however, Experiment 3 revealed contrasting findings. This study provides systematic behavioral evidence for the “dual processing” hypothesis of fraction cognition through three experiments. Our data indicate that the spatial representation of fractions does not rely on a single path but involves two information processing systems: overall value and component size, and its behavioral expression is highly regulated by cognitive control. When the overall value of the fraction and the component values are clear and not in conflict, we observed a stable attentional SNARC effect. The rapidity and task independence of this effect strongly support that numerical values can be rapidly extracted and mapped onto spatial attention. Experiment 3 employed a critical conflict design (where the overall value was contrary to the component size information). Under this condition, any single-pathway automatic activation should have produced observable behavioral effects (either conflict or dominance of one side), but what we observed was the systematic disappearance of all effects at the behavioral level. The most concise and powerful explanation for this “zero result” is that both the overall and component information were deeply processed and generated internal conflicts. This conflict immediately triggered a top-down cognitive control mechanism (conflict monitoring and active inhibition), which prevented the spatial signals of the conflict from influencing behavior, thereby ensuring the efficiency of the current task execution. The above results indicate that “overall and component value processing” is not a static, all-or-none phenomenon, but rather a dynamic and context-dependent collaborative process. Under normal conditions, when overall and component value information are consistent, they are rapidly extracted and spatially mapped; however, when fine-grained analysis is required or conflicting information arises, cognitive control mechanisms are engaged to maintain task efficiency and accuracy.

### 5.1 Association between fraction magnitudes and spatial representation

In the three experiments, different types of numerical sets were presented as cue stimuli. The results of experiments 1 and 2 indicate that the processing of fractions elicited a shift in spatial attention. This outcome suggests an association between ratio processing and spatial representations that can produce an attentional SNARC effect. The results of experiments 1 and 2 denote that there is a psychological number line in the participants' mental representations associated with the numerical values or components of fractions. Larger numerical values tend to elicit a rightward shift in spatial attention, whereas smaller numerical values lead to a leftward shift in spatial attention. Previous studies have obtained comparable results across diverse experimental tasks (Fischer et al., 2003; Dodd et al., 2008). Experiments 1 and 2 revealed that fraction processing can automatically trigger spatial attentional shifts. However, in the “congruent condition,” the overall value and component values of the fraction predict the spatial direction in the same way, so the observed effect cannot distinguish the underlying driving source. To clarify whether it is the overall value, the component value, or the combined effect of both that triggers the attentional shift, we designed Experiment 3. This experiment introduced a crucial “conflict condition” where the overall value and component values predict the direction of attention in opposite ways, thereby conducting a competitive test of the above possibilities. In Experiment 1, a significant difference in RTs for detecting stimuli presented on the left and right sides was observed when the CTI was set to 250 and 500 ms. In Experiment 2, we noted significant differences in RTs for both sides when we set the CTI values to 500 ms and 750 ms. The findings from Experiments 1 and 2 demonstrate a statistically significant disparity in response speed to stimuli between the left and right sides, with varying latency periods required for this divergence. Experiment 1 highlighted that the initiation of attentional shifts requires a certain amount of time; however, Experiment 2 revealed differences in the processing mechanisms associated with various types of numerical materials, which may involve differing demands on attentional resources.

The attentional SNARC effect observed in this study demonstrated significant fragility and time-dependency: it only reliably emerged at a specific cue-target interval (CTI, such as 250 ms), and weakened or vanished under longer CTIs or conflict conditions. This pattern provides a novel mechanistic framework for understanding the key controversy surrounding the replicability of the attentional SNARC effect, as illustrated by Colling et al. (2020), who failed to replicate the effect in a large-sample, multi-laboratory replication effort. We suggest that the sensitivity of the effect in this study to precise experimental parameters (such as processing depth, CTI, etc.) may be a key reason why some previous studies failed to replicate it successfully. Therefore, our findings do not negate the attentional SNARC phenomenon itself, but rather precisely delineate its boundary conditions and emphasize the importance of strictly controlling the temporal process in methodology for capturing such rapid extraction and processing. Furthermore, this “fragility” precisely reveals that the attentional SNARC effect is not a rigid, consistent response, but a dynamic cognitive process. This shifts the focus in the field from merely debating “whether the effect exists” to a more ecologically valid and theoretically significant question of “under what conditions the effect emerges and how it is modulated.”

### 5.2 The dynamics of fraction processing: evidence of co-activation from conflict effects

The representation of fractions is the subject of ongoing debate. Mental representations of fractions can be categorized as holistic, component, or hybrid. We employed the target detection paradigm to investigate whether an attentional SNARC effect exists during the processing of fraction magnitude comparisons. We aimed to verify whether the spatial representation of fractions would activate both a fraction's overall value and the magnitudes of its components. The findings from the three experiments suggest that the processing and representation of fractions elicit concurrent activation of both the magnitude of the fraction itself and the magnitudes of its constituent parts. Based on this, we propose that the processing and representation of fractions simultaneously employ two representational strategies (i.e., holistic and componential), which is in line with previous studies. Meert et al. (2009) found, in an adult number comparison task, that fraction magnitude is mentally represented in a hybrid format—combining both componential and holistic representations. Another study utilized computer mouse-tracking technology to investigate the process of fraction comparison. The results imply that individuals simultaneously utilize both component and holistic representations; however, there is a continuum and competition between them (Faulkenberry et al., 2015). Specifically, if a task requires processing only the integer components of the fractions to arrive at a solution, individuals may prioritize component strategies. Conversely, if a task requires integrating two integer components to make an accurate judgment, individuals are likely to adopt holistic strategies. When processing fractions, individuals select appropriate cognitive strategies based on the tasks and stimuli presented (Schneider and Siegler, 2010). Rivera and Soylu (2021) noted that in addition to employing full component-based or holistic processing representations, participants may select generalized fraction strategies based on the demands of the task. Individuals employ a mixed mental representation during the comparison process, which encompasses both components and holistic representations. This process is influenced by a phenomenon known as whole-number bias because individuals tend to rely on principles of integer comparison when assessing the magnitude of fractions, which may lead them to habitually activate the corresponding whole number during the processing and representation of fractions (Abreu-Mendoza et al., 2023; Leib et al., 2023).

Based on behavioral data, this study suggests that there might exist a “conflict monitoring - proactive inhibition” pattern, which is also consistent with the findings of existing neuroscientific research. For example, Zhang et al. (2012) discovered that, according to the results of event-related potential (ERP), in the simple condition, there was a distance effect of component processing in the P3 component, but in the complex condition, the result was not the same. Neurological research on the processing of fractions suggests that the processing of fractions may not rely on a single processing strategy but may require the adoption of an inhibitory strategy to counteract the misleading influence of whole number bias. Poirel et al. (2012) found in an functional magnetic resonance imaging (fMRI) study that in tasks similar to Piaget's conservation of number task, people rely on the activation of right-brain related brain regions to suppress misleading strategies. Therefore, this study, through a meticulous behavioral experimental design, revealed a possible rapid inhibitory control process in fraction processing at the behavioral level and provided indirect but systematic behavioral evidence for it. Future research can utilize techniques such as fMRI and ERP to directly verify whether the prefrontal-parietal control network is specifically activated under the conflict paradigm of this study and its dynamic changes over the processing time course. Thus, the behavioral model of this study provides clear theoretical predictions and an ideal behavioral paradigm for subsequent cognitive neuroscience research.

Previous studies (e.g., Meert et al., 2009; Faulkenberry et al., 2015) have proposed that holistic and componential representations can coexist or compete. The key advancement of this study lies in the use of an attentional paradigm (rather than explicit comparison tasks) and a conflict design, which enables us to examine how this coexistence/competition is resolved in a non-strategic, rapid time window. Our findings suggest that this “competition” is not a static trade-off but a dynamic, controlled process: when information is consistent, a certain representation (such as component values) can rapidly drive spatial attention (Experiments 1 and 2); when conflict is detected, an immediate inhibitory control mechanism is activated to actively arbitrate and mask the spatial signals of the conflict (Experiment 3). This shifts the discussion from “whether there are two representations” (what) to the mechanism level of “how the brain manages and regulates the conflict output of these two representations in real time” (how). In summary, this study has advanced the research on hybrid fraction representations by combining a time-resolved attentional paradigm with a diagnostic conflict design.

### 5.3 Limitations and future directions

#### 5.3.1 Constraints of the stimulus set and generalizability

The limitation of this study lies in the fact that only four fractions were used as stimulus materials in each experiment. This focused design, while allowing us to strictly control and repeatedly measure key theoretical conditions such as numerical consistency and whole-part conflict, inevitably restricts the generalizability of our research conclusions. Our findings (such as the rapid numerical extraction effect and conflict-induced inhibition) are most directly applicable to fractions that are similar in numerical structure and contrast relationship to the experimental materials. It is currently not possible to assert whether and how these mechanisms extend to more complex fractions (such as mixed numbers and improper fractions) or a broader range of numerical values. This design was a conscious trade-off made by us in the initial exploratory stage of the research. We prioritized the fractions that were theoretically most diagnostic (for example, using 8/16 to cleanly separate the whole value from the component size to create a “conflict” condition), thereby testing the core theoretical construct (the competition between whole and component representations) in the most efficient way. This helped to obtain clear preliminary evidence in terms of internal validity. Future research urgently needs to systematically expand the fractional stimulus set to validate and extend the conclusions of this study. Specific directions include: (1) Increasing the number and range of stimuli: incorporating a more diverse set of fractions to examine the robustness of the current effects and their relationship with numerical properties of fractions (e.g., numerator-to-denominator ratio, magnitude); (2) Directly testing the boundary conditions of competing hypotheses: designing experiments to directly compare the applicability of “holistic processing advantage” vs. “componential processing advantage” across distinct experimental contexts.

#### 5.3.2 Methodological considerations and confounding variables

A key methodological consideration of this study needs to be pointed out: the core theoretical dimensions we focused on (overall value and component size) and the potential confounding variables (familiarity and perceived complexity) are inherently covariant in the stimulus materials. For instance, a fraction with a large overall value (such as 18/19) necessarily contains large component numbers, and its visual complexity and familiarity typically differ from those of a simple fraction (such as 1/2). This intrinsic association makes it extremely difficult to manipulate these attributes completely orthogonally in behavioral experiments. Therefore, although the effect patterns we observed are highly consistent with the theoretical predictions of the “overall-component” representation conflict, the potential contribution of low-level perception or familiarity differences cannot be absolutely ruled out. Nevertheless, this study aims primarily to conduct an initial theoretical test, not to comprehensively map all contributing factors. To this end, we adopted a diagnostic paradigm: specifically, we selected stimuli that, according to theory, most sharply isolate the target contrast (e.g., 8/16, which most purely instantiates the “conflict” between a small overall magnitude and large component magnitudes). Although this strategy entails accepting variable co-variation as a trade-off, it maximizes our ability to detect the systematic conflict effect within the spatial attention task. Future research must move beyond this limitation through more refined experimental designs. One promising solution is to construct and standardize a large, normed stimulus library of fractions, with pretested and quantified estimates of familiarity, visual complexity, and other relevant perceptual attributes. Building upon such a resource, researchers could: (1) statistically control for these confounding variables (e.g., by including them as covariates in regression or mixed-effects models); (2) design experiments enabling near-orthogonal manipulation of these attributes; and (3) integrate computational modeling to quantify the unique variance in behavior attributable to each attribute. Such advances would robustly propel the field from mere detection of effects toward precise mechanistic characterization.

#### 5.3.3 Statistical power and sample size

The sample size of this study (N ≈ 27-29 for each experiment) was determined based on a priori power analysis aimed at detecting the main effects (such as the attentional SNARC effect), providing sufficient statistical power (> 0.80) for a medium effect size (f = 0.25) as expected. This design ensured the reliability of key findings, such as the significant spatial attention effect in Experiments 1 and 2. However, we acknowledge that the relatively limited sample size may have affected the ability to detect more complex effects (such as higher-order interactions) and may have restricted the overall robustness of the study's conclusions. Although the observed behavioral patterns were clear and theoretically consistent, a larger sample size would undoubtedly provide more precise estimates of effect sizes and enhance the power to test potential moderating effects. Bayesian analysis for null results: For the core finding in Experiment 3 that “the effect disappears under conflict conditions,” we not only reported the non-significant results of frequency statistics but also calculated the Bayes factor. The result (BF10 = 0.117) provided “strong” evidence in support of the null hypothesis (H0, i.e., no effect), which to some extent compensates for the limitations of traditional statistics in supporting the conclusion of no difference. Reporting of effect sizes: We reported the effect sizes of the main comparisons (such as Cohen's d) in the results, which helps readers assess the magnitude of the effects rather than relying solely on significance tests. Future studies should replicate this experiment with a larger sample size to confirm the replicability of the current effects and more effectively explore possible individual differences or situational moderating factors.

#### 5.3.4 Integrated future research directions

Building on the present findings—and acknowledging their inherent limitations—future research should advance this line of inquiry along several interconnected directions. First, although the current stimulus set was selected for high theoretical diagnosticity, its generalizability remains limited. Future studies should systematically broaden the stimulus range—for example, by incorporating improper fractions and mixed numbers spanning a wider numerical continuum—and adopt experimental designs that orthogonally manipulate overall magnitude, component magnitudes, familiarity, and visual complexity. The development of a standardized, rigorously quantified fraction stimulus library is essential to isolate the unique contributions of these factors and support stronger causal inferences regarding the competition between holistic and componential representations. Second, beyond large-scale replications to establish robustness, applying this paradigm to individuals with varying levels of mathematical proficiency (e.g., high vs. low fractional competence), across distinct developmental stages (e.g., children, adolescents), and among educators (e.g., teachers) will critically test the universality of the observed effects and elucidate how hybrid representational systems and conflict-monitoring mechanisms are shaped by experience and cognitive development. Finally, to move beyond the indirect inferences afforded by behavioral data and to directly test the proposed dynamics of conflict monitoring and inhibitory control, the integration of high-temporal-resolution EEG/ERPs with high-spatial-resolution fMRI constitutes a critical methodological advance. Such multimodal neuroimaging approaches precisely characterize the spatiotemporal dynamics of these cognitive processes—identifying when and where they occur in the brain—and thus provide decisive empirical support for the dual-process model of rapid extraction and adaptive inhibition.

### 5.4 Implications for mathematics education

Based on the findings of this study, we propose several possible teaching suggestions: (1). Utilize the “time window” of conflict for teaching: Our data indicate that the inhibitory control triggered by conflict occurs in the later stage after the presentation of the stimulus. This suggests that when students are initially learning fractions, slowing down the teaching pace and providing sufficient “wait time” at critical decision points (such as when comparing the sizes of fractions) may help the cognitive control mechanism to intervene, thereby overcoming the interference of the automatic integer bias. (2). Cultivate metacognitive awareness of “conflict”: Teachers can explicitly guide students to identify and mark the internal conflicts they may encounter (for example, “the numerator increases, but the overall fraction value decreases”). By making such implicit conflicts explicit and practicing pausing and re-evaluating when conflicts occur, students' monitoring and regulation abilities may be enhanced. (3). Design “conflict-adaptive” exercises: Teaching materials can be consciously arranged to present consecutive pairs of conflicting and non-conflicting fractions (such as comparing 1/3 vs. 4/9 first, and then 3/7 vs. 8/9). This design may simulate and strengthen the adaptive inhibition process found in the experiment, helping students develop more flexible and controlled fraction processing strategies. In conclusion, the dynamic nature of the fraction space mapping and the conflict monitoring mechanism revealed in this study provide a fine-grained cognitive process perspective for understanding difficulties in fraction learning. Future educational intervention research can build on this foundation to develop and test teaching strategies that aim to optimize the application of cognitive control resources rather than merely repeating conceptual explanations.

## 6 Conclusions

This study demonstrates that spatial attention mapping for fractions is a dynamically regulated process. Our findings provide empirical support for a dual-process mechanism: the parallel activation of holistic and componential fraction representations triggers conflict monitoring and recruits adaptive inhibitory control. Consequently, this work reframes numerical–spatial associations—not as a static representational phenomenon, but as a time-dependent process governed by cognitive control. Future neuroimaging studies will be instrumental in elucidating the neural substrates and spatiotemporal dynamics underlying these interacting cognitive pathways.

## Data Availability

The original contributions presented in the study are included in the article/supplementary material, further inquiries can be directed to the corresponding author/s.
